# The Effects of Collagen Peptides as a Dietary Supplement on Muscle Damage Recovery and Fatigue Responses: An Integrative Review

**DOI:** 10.3390/nu16193403

**Published:** 2024-10-08

**Authors:** Pedro Augusto Querido Inacio, Yasmin Salgado Mussel Gomes, Ana Julia Nunes de Aguiar, Pedro Sardinha Leonardo Lopes-Martins, Flávio Aimbire, Patrícia Sardinha Leonardo, Alberto Souza Sá Filho, Rodrigo Alvaro B. Lopes-Martins

**Affiliations:** 1 Laboratory of Applied Neurosciences, Evangelical University of Goiás, UniEvangélica, Av. Universitária S/N, Anápolis P.O. Box 75083-515, GO, Brazil; pedroqinacio@gmail.com (P.A.Q.I.); alberto.filho@unievangelica.edu.br (A.S.S.F.); 2 Laboratory of Biophotonics and Experimental Therapeutics, LABITEX, Evangelical University of Goiás, UniEvangélica, Av. Universitária S/N, Anápolis P.O. Box 75083-515, GO, Brazil; yasminmussel@gmail.com (Y.S.M.G.); pdlopesmartins@gmail.com (P.S.L.L.-M.); 3 Translational Medicine, Federal University of São Paulo-UNIFESP, São José dos Campos P.O. Box 12247-014, SP, Brazil; flavio.aimbire@unifesp.br; 4 Laboratory of Health Technologies, LATES, Evangelical University of Goiás, UniEvangélica, Av. Universitária S/N, Anápolis P.O. Box 75083-515, GO, Brazil; patyssardinha@gmail.com; 5 Post-Graduate Program in Bioengineering, University Brasil, Av. Carolina Fonseca 236, Itaquera, São Paulo P.O. Box 08230-030, SP, Brazil

**Keywords:** collagen peptides, hydrolyzed collagen, muscle recovery, muscle mass

## Abstract

Background/objectives: The oral administration of hydrolyzed collagen peptides is a scientifically validated intervention for enhancing skeletal muscle health and performance. This integrative review consolidates the evidence supporting the use of low molecular weight collagen peptides (2000–3500 daltons) for their superior bioavailability and absorption. Our objective was to review the effects of collagen peptide or hydrolyzed collagen supplementation on muscle damage, recovery, and construction related to physical exercise. Methods: A bibliographic search was conducted in major English-language databases, including PubMed/Medline, using terms like “Peptides Collagen and Damage” and “collagen peptides AND Soreness Muscle”. This review followed PRISMA guidelines, with bias risk assessed via the PEDro scale. The inclusion criteria were (a) randomized clinical trials, (b) randomized studies in humans with a control or placebo group, (c) studies assessing muscle damage or delayed onset muscle soreness via physiological markers or strength performance tests, and (d) studies using hydrolyzed collagen or collagen peptides. Results: Initially, 752 articles were identified. After applying the inclusion and exclusion criteria, including duplicate removal, eight articles with 286 participants were included. Of these, 130 participants received collagen peptide supplementation, while 171 received a placebo or control. Conclusion: This integrative review supports the potential of collagen peptide supplementation to mitigate muscle stress from acute strenuous resistance training. However, due to the methodological heterogeneity among the studies, further clinical trials are needed to clarify the mechanisms underlying muscle improvement with collagen supplementation.

## 1. Introduction

The regular practice of physical exercise is emphasized across various health promotion guidelines by numerous organizations [1]. The benefits of maintaining an active lifestyle are well-documented, particularly concerning one’s health and quality of life [2,3,4]. However, for those unaccustomed to such practices, the initial engagement in various training programs often presents a significant barrier [5,6]. Muscle damage scenarios or delayed onset muscle soreness (DOMS) can emerge within hours after or upon completion of the activity, persisting for up to 7 days [7,8]. Aside from the discomfort experienced by new participants, a comprehensive review highlights that the first few weeks of resistance training and the potential muscular adaptations primarily involve tissue repair [8], with outcomes such as hypertrophy being evident only after 18 sessions.

### 1.1. Collagen and Muscle Mass

Hydrolyzed collagen supplementation has gained significant attention in the scientific literature for its positive effects on skeletal muscle and the enhancement of muscle mass. In 2015, Zdzieblik et al. [9] investigated collagen peptide supplementation in combination with resistance training showing that the treatment improved the body composition and increased the muscle strength in elderly sarcopenic men.

Recently, studies have demonstrated that the intake of hydrolyzed collagen peptides can significantly improve muscle function and promote hypertrophy, particularly in individuals engaged in resistance training [10]. The authors demonstrated that the use of resistance exercise training in combination with collagen peptide supplementation resulted in a more pronounced increase in body mass, muscle mass, and muscle strength than exercise alone. This benefit is attributed to the high bioavailability of collagen peptides, which are rich in essential amino acids such as proline and glycine, critical for muscle tissue repair and growth. Notably, the efficacy of collagen peptides is closely linked to their molecular weight, with peptides in the range of 2000 to 3500 daltons showing superior absorption and effectiveness compared to those with a molecular weight of around 5000 daltons. These findings underscore the potential of hydrolyzed collagen as a valuable intervention for improving skeletal muscle health and supporting muscle mass gain.

In terms of practical applications, the potential benefits of collagen peptide supplementation may vary depending on the population in question. For athletes engaged in high-intensity training, collagen peptides could support muscle recovery by reducing markers of muscle damage and accelerating strength recovery post-exercise. This is particularly relevant in reducing the downtime between training sessions and improving the overall athletic performance. On the other hand, elderly populations, who naturally experience a decline in collagen synthesis and muscle mass due to aging, may benefit from collagen supplementation in terms of improving muscle mass retention and function. By promoting muscle repair and enhancing physical strength, collagen peptides could play a crucial role in preventing age-related muscle degeneration and improving the quality of life in older adults. This suggests that tailored supplementation strategies, considering factors such as age, activity level, and individual health status, are essential for optimizing the therapeutic benefits of collagen peptides.

### 1.2. Muscle Damage and Recovery

Although muscle damage remains a topic of debate due to its multifaceted etiology [11,12,13], indirect physiological markers have been utilized, such as curves of lactate dehydrogenase (LDH), creatine kinase (CK), myoglobin, and *C*-reactive protein (Brancaccio et al., 2010) [14]. In addition to these biomarkers, acute reductions in field tests involving force production, whether maximal or across other spectrums such as endurance strength (maximal voluntary isometric contractions), are considered reliable indicators of potential muscle damage [15,16]. To address the issue of delayed onset muscle soreness, which empirically may be considered a barrier to training adherence, the pharmaceutical and fitness industries have been developing potential solutions that do not compromise strength gains or muscle growth [17,18].

It is noteworthy that the literature already partially supports physical and manual treatments that appear to mitigate delayed onset muscle soreness, as well as supplemental sources rich in proteins [19,20,21]. However, the focus of this review is on supplementation, without excluding manual interventions. Among the beverages considered to aid in muscle damage repair or attenuation, collagen peptide supplementation has gained prominence in clinical studies [22,23]. Collagen peptides (CP) or hydrolyzed collagen are known for their good bioavailability to human tissues, characterized by a low molecular weight and richness in amino acids such as proline and hydroxyproline [24]. Some investigations have reported potential positive effects from CP use, including reductions in delayed onset muscle soreness, improvements in countermovement jump tests, and maximal voluntary isometric contraction tests [25,26].

Despite these promising findings, the literature still lacks a comprehensive review of the evidence, one that summarizes the evidence and clarifies the quality and potential directions for new clinical trials on CP supplementation and its effects on muscle damage and fatigue resulting from physical training. This is the focus of the present investigation.

## 2. Materials and Methods

### 2.1. Search for Periodicals and Review Process

A search was conducted for clinical trial publications in English within the main databases, including PubMed/MEDLINE, Scopus, Web of Science, and Cochrane Library. This review was constructed and conducted according to the Preferred Reporting Items for Systematic Reviews and Meta-Analyses (PRISMA) guidelines. We also consulted the database for ongoing reviews (PROSPERO). The search process occurred from July 2024 to the first week of September 2024 and was conducted by two independent researchers (P.I) (R.L.M).

### 2.2. Inclusion and Exclusion Criteria

Our analyses were limited to peer-reviewed periodicals that published articles in English meeting the following criteria: (a) randomized clinical trials, (b) randomized studies in humans that included a control or placebo group, (c) research investigating muscle damage or delayed onset muscle soreness using physiological markers or field tests evaluating strength performance, and (d) studies utilizing hydrolyzed collagen or collagen peptides. Review articles, brief reviews, opinion articles, case studies, or observational studies, as well as research using non-hydrolyzed forms of collagen or that did not employ any exercise protocol designed to induce acute muscle damage, were deemed ineligible for inclusion in this review.

### 2.3. Search Strategy

**Figure 1:**
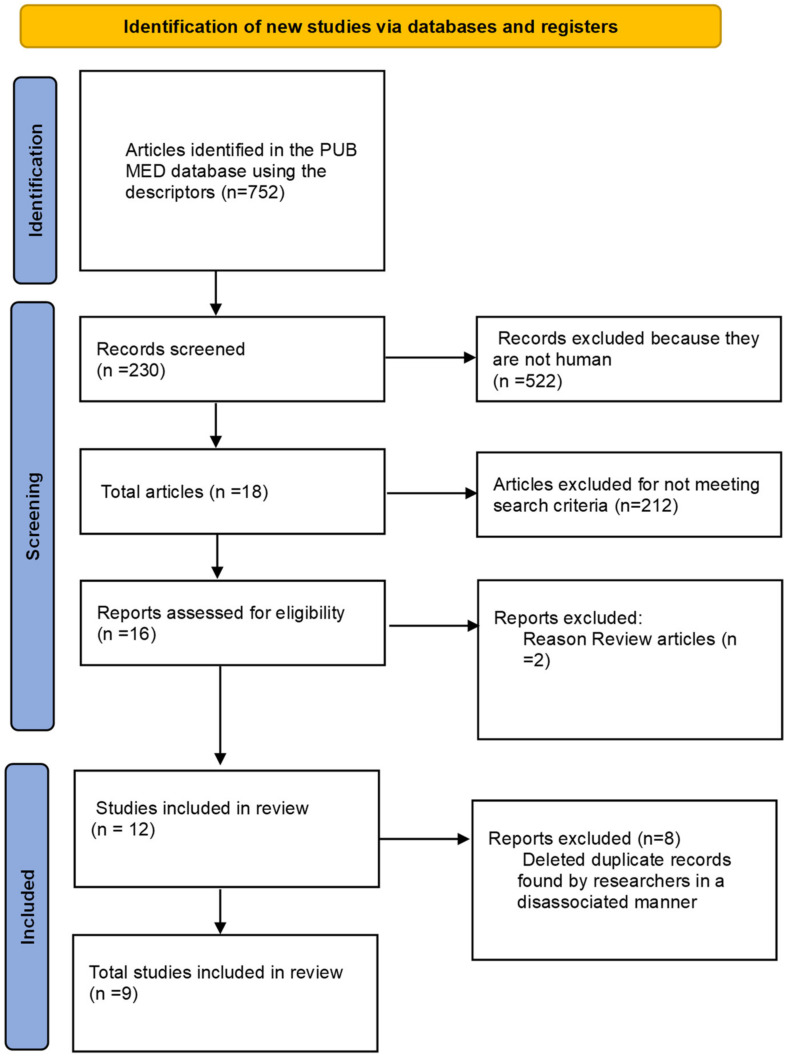
PRISMA Flowchart. Database articles included and excluded in review.

The following syntax was used to perform the search: collagen peptides AND muscle damage and collagen peptides AND soreness muscle. Articles that contained any of the following terms in their title or abstract were included for further analysis: collagen hydrolyzed, muscle damage, low molecular weight collagen, creatine kinase, recovery, collagen peptides, muscle recovery, exercise-induced muscle damage, and fatigue response. Among these selected articles, the reference lists were subsequently reviewed to identify any additional relevant publications [27]. To avoid selection bias, the search was conducted by four researchers who independently analyzed the articles. In cases of disagreement regarding inclusion or exclusion, a fifth researcher was consulted for the final decision. Figure 1 shows the PRISMA flowchart of papers selections.

### 2.4. BIAS Evaluation

To assess the risk of bias, the Physiotherapy Evidence Database (PEDro) scale was used as a tool. The scores and their respective classifications are as follows: scores < 4 are considered “poor”, 4–5 is considered “fair”, 6–8 is considered “good”, and 9–10 is considered “excellent” [28]. P.I and A.J.N.A conducted the methodological evaluation of the final included studies. A third reviewer (A.S.F.) resolved any disagreements, if necessary. The scores from the selected articles, were addressed through the PICO model (Population: age range, sex, untrained or trained), (Intervention: protocol used to induce muscle damage and fatigue scenarios) (Control: type of placebo used and collagen peptides when specified) (Outcomes: Results).

## 3. Results

From the studies searched in the database, 752 articles were identified. After applying filters and exclusion criteria, 522 investigations were discarded as they did not involve human studies, resulting in 230 articles that were selected for a thorough evaluation, including the reading of the title and abstract by independent researchers. After applying the inclusion and exclusion criteria, 16 manuscripts remained, of which two were excluded, as they were reviews covering other potential effects of collagen peptide supplementation. Since the research was conducted by two independent researchers, duplicated articles were included for the final analysis, with these duplicates being subsequently excluded. This process resulted in a total of eight articles that met the criteria for this investigation, and their results are presented in Table 1.

**Table 1:** Selected Articles from Systematic Review on Collagen Supplementation for Muscle Improvement.

Author; Country; PEDro Score	Population	Type Training	Groups	Outcomes	Results
[21]; Austria Pedro 10	N = 55 Sex male 18–40 years; untraineds	concurrent training (CT) 3/week 12 weeks	15 g placebo (n = 29) 15 g CP (n = 26)	Maximum voluntary contraction (MVC), rate of force development (RFD) peak RFD, Countermovement jump (CMJ) and muscle soreness (MS) which was measured from visual analog scale (VAS) with numbers ranging from 0 mm (no pain) to 100 mm (unbearable pain).	As a between-subjects factor and testing time (pre, post, 24 h, 48 h) and study time (T1, T2) as within-subject factors. Significant 3-way interaction effect for MVC *p* = 0.02. ΔRFD = (*p* < 0.01); CMJ = (*p* = 0.046); MS = (*p* = 0.66). The results indicate a positive effect for PC supplementation; however, no differences were reported for the effect of muscle soreness from the VAS.
[25]; UK Pedro 10	N = 24 Sex male; 24 ± years; recreationally active	Acute intervention (drop jump)	20 g placebo (n = 12) 20 g CP (n = 12) + bebeida enriquecida com 80 mg de vitamina C	Maximum isometric voluntary contraction (MIVC), Countermovement jump (CMJ) and muscle soreness (MS) measured as both subjective pain and pressure pain threshold (PPT) which was measured from visual analog scale (VAS) with numbers ranging from 0 mm (no pain) to 200 mm (unbearable pain), blood sampling(CK, AST, ALT, LDH, IL-6, β-NGF), Bone turnover markers.	MS = time effect for increased was observed (*p* = 0.001), but no time × group interaction (*p* = 0.202), PPT = CP was for reducing soreness at 24 h post-exercise (CP 106.67 ± 43.98 mm vs. CON 139 ± 35.68 mm) and likely beneficial at 48 h post-exercise (CP 90.42 ± 45.33 mm vs. CON 125.67 ± 36.50 mm). CMJ = height at 24 h post-exercise (CP 86.65 ± 11.94% vs. CON 79.69 ± 12.64% of baseline values; ES = 0.33), MIVC = effect time in both groups reduction (*p* = 0.001) but no time × group (*p* > 0.05) 24 h post-exercise, was 85.35 ± 15.77% of baseline values in CP and 78.44 ± 17.7% in CON. AST and CK ↑48 h in CON (*p* > 0.05).
[22]; Austria Pedro 10	N = 55 Sex male 18–40 years; untraineds	concurrent training (CT) 3/week 12 weeks	15 g placebo (n = 29) 15 g CP (n = 26)	Creatine kinase (CK), lactate dehydrogenase (LDH), myoglobin (MYO) and high-sensitivity C-reactive protein (hs-CRP) were analyzed before, after and 2 h, 24 h and 48 h after exercise.	Analysis of the area under the curve shows significant differences with a smaller increase in MYO (*p* = 0.004, ηp2 = 0.184) CK (*p* = 0.01, ηp2 = 0.145) and LDH (*p* = 0.016, ηp2 = 0.133) in group CP. Differences in absolute averages showed significant difference in effect size MYO (*p* = 0.017, d = 0.771), CK (*p* = 0.039, d = 0.633) e LDH (*p* = 0.016, d = 0.764) by CP supplementation.
[29]; Belgium Pedro 11	N = 22 Sex male years; recreationally active	(RT) knee extension, one-leg-squat, dropjumps 17 training sessions/week 3 weeks	Control 45 g whey protein W (n = 11) 25 g whey protein + 20 g WCP (n = 11)	Dynamic maximal voluntary contraction ((MVC_dyn_),an isometric maximal voluntary contraction test (MVC_iso_), a countermovement jump test (CMJ), as well as a 25-repetition maximum test (25-RM) on a knee extension device. *N*-terminal procollagen peptide type 1.	25-RM test in group W ↑10% pre (21.6 ± 3.9 kg) post (23.7 ± 5.6 kg); WCP↑22% pre (20.3 ± 5.2 kg) post (24.8 ± 5.6 kg, P_time_ < 0.001). Total weekly workload W↑32% and WCP ↑%. Increases in serum concentration of *N*-terminal procollagen peptide type 1 by 10% (Ptime < 0.01). However, no differences were found for any of the outcomes between W and WCP
[30] Oklahoma Pedro 9	N = 15 Sex male years; trained	15 g CP (n = 7) PLA (n = 8)		Resistance-trained males consumed 15 g/day of CP (n = 7) or placebo (n = 8), and after 7 days, maximal voluntary isometric contraction (MVIC), countermovement jump height, soreness, and collagen turnover were examined.	CP supplementation attenuated performance decline 24 h following muscle damage. Acute consumption of CP may provide a performance benefit the day following a bout of damaging exercise in resistance-trained males
[31] California Pedro 11	N = 50 Sex male, active collegiate athletes 18–25 years	Power training three times per week 3 weeks	HC20 g + C 50 mg (n = 23) Placebo 20g (n = 25)	Rate of force development (RFD), Countermovement jump (CMJ)	HC + C group demonstrated a subsequent recovery of RFD to the baseline value by Test 3 (*p* = 0.07) both groups increased maximum isometric force (PLA = 7.09 ± 2.80%; HC + C = 7.81 ± 2.60%) RFD decreased in the PLA group (−16.20 ± 4.00%) and was not different from zero in the HC + C group (−2.13 ± 5.20%). CMJ jump height shows no difference between groups.
[23], Netherlands Pedro 11	N = 45 Both male and female sex, age 21–29, trained	Acute intervention (squat training)	30 g WHEY protein (n = 15) 30g collagen protein (n = 15) Placebo (n = 15)	Muscle soreness (MS) was mesured with Likert Scale for pain with a score of 0 indicating complete absence of soreness and 6 indicating severe pain that limits the ability to move.	For the three groups Whey, placebo and collagen there was no significant difference *p* > 0.5 on the Likert scale.
[10], UK Pedro 10	N = 53 Male, Sex age 72, untraineds	Acute intervention	15 g collagen peptides 15 g placebo	Fat mass, Free-fat mass, Body mass, bone mass.	collagen peptide supplementation in combination with resistance training further improved body composition by increasing FFM, muscle strength and the loss in FM
[26], Japan Pedro 10	N = 20 Male, Sex age 40–65, untraineds	Acute intervention (squat training)	5 g collagen peptides (n = 10) 5 g placebo (n = 10)	Muscle soreness (MS), muscle fatigue (MF) and muscle strength were mesured with visual analog scale (VAS) with numbers ranging from 0 mm (no pain) to 200 mm (unbearable pain), blood sampling (CPK and LDH).	VAS muscle soreness collagen group (32.0 ± 25.0 mm), placebo group (45.8 ± 27.6 mm), Cohen’s d 0.678. VAS fatigue collagen group (47.3 ± 25.1 mm), placebo group (59.0 ± 22.3 mm), Cohen’s d 0.715. muscle strength collagen group (85.2 ± 27.8 kg), placebo group (80.5 ± 25.3 kg) on day 3, −4.8 kg, 95% CI: −9.3~−0.2 kg, Cohen’s d = 0.460. No significant difference in ROM. CPK and LDH did not show significant differences, only for the time factor (base line, day 3) *p* < 0.001.

### 3.1. Population

A total of 339 participants were included in the interventions, of whom 157 received some type of collagen intake intervention, and 195 received placebo. One study combined vitamin C and collagen supplementation [31], and another investigation combined the use of collagen peptides and whey protein [30]. Among the studies investigated, most utilized healthy individuals who were not regular practitioners of strength training or aerobic conditioning, thus ensuring their detrained status. One article included elite athletes at the university level [31], and another study focused on middle-aged adults of both sexes [26].

### 3.2. Body Composition

Oertzen-Hagemann [10] investigated the effects of collagen peptide supplementation combined with resistance exercise training (RET) on skeletal muscle protein composition in young men. Twenty-five participants (average age 24.2 years) completed a 12-week full-body hypertrophy workout program, with half receiving 15 g of collagen peptides daily and the other half receiving a non-caloric placebo. Both groups showed significant increases in body mass (BM) and fat-free mass (FFM), with the collagen group (COL) showing more pronounced gains compared to the placebo group (PLA). Strength improvements were also higher in the COL group. Proteomic analysis revealed that 221 proteins were more abundant in the COL group, particularly those associated with the metabolism of contractile fibers, compared to only 44 proteins in the PLA group. These findings suggest that collagen peptide supplementation enhances the effects of RET on muscle mass, strength, and protein composition, particularly in contractile fibers.

Zdzieblik et al. [9] in a randomized, double-blind, and placebo-controlled study investigated the impact of post-exercise collagen peptide supplementation on muscle mass and strength in elderly men with sarcopenia. Fifty-three participants (average age 72.2 years) underwent a 12-week resistance training program, receiving either 15 g/day of collagen peptides (treatment group) or silica as a placebo (placebo group). The results showed significant improvements in the fat-free mass (FFM), bone mass (BM), isokinetic quadriceps strength (IQS), and sensory motor control (SMC) across all participants, with greater effects observed in the collagen peptide group. Specifically, the treatment group experienced more pronounced gains in FFM (+4.2 kg vs. +2.9 kg) and IQS (+16.5 Nm vs. +7.3 Nm) and a greater reduction in fat mass (FM) (−5.4 kg vs. −3.5 kg) compared to the placebo group. These findings suggest that collagen peptide supplementation, combined with resistance training, enhances muscle mass, strength, and body composition more effectively than resistance training alone.

### 3.3. Rate of Force Development and Reduction

Among the markers of force development, including countermovement jump (CMJ) and maximal voluntary isometric contractions, the studies support the use of collagen peptides (CP) compared to placebos in interventions aimed at inducing muscle damage scenarios and evaluating the deleterious effects on force production. Clifford et al. [24] showed that the CMJ height recovered more quickly in the CP group compared to the placebo group (*p* = 0.050, ES = 0.55) relative to the baseline values. A study on recreationally trained individuals showed a positive effect for the 25-repetition maximum knee extension test, favoring the group supplemented with a combination of collagen peptides and whey protein, with a 22% increase in repetitions compared to a 10% increase in the whey-only group [31]. Regarding the maintenance of the rate of force development, positive effects were also reported by Kuwaba et al. [26] and collaborators, where the collagen group experienced a smaller reduction in maximal strength with a moderate effect size (Cohen’s d = 0.460).

### 3.4. Biochemical Markers

Of the eight studies included in this review, only three articles evaluated markers related to potential muscle fatigue and damage scenarios, including creatine kinase, lactate dehydrogenase (LDH), interleukin-6, and myoglobin [22,24,25]. All protocols were sufficient to acutely increase these markers, indicating a possible scenario of increased systemic muscle stress. However, the results of Clifford et al. and collaborators [24] regarding blood markers did not show significant differences (*p* > 0.05). Contrasting these findings, Bischof et al. [22] demonstrated that 12 weeks of CP supplementation in untrained men resulted in significantly smaller increases in myoglobin, creatine kinase (CK), and LDH markers. In terms of absolute means, medium effect sizes were observed for the CP group: CK (*p* = 0.039, d = 0.633), LDH (*p* = 0.016, d = 0.764), MYO (*p* = 0.017, d = 0.771). Aligning with Clifford’s results, Kuwaba et al.‘s investigation also did not report significant differences in physiological markers for a muscle stress task supplemented with collagen peptides versus a placebo, where the CPK and LDH did not present significant differences, except for the time factor (baseline, day 3) (*p* < 0.001).

In reviewing the studies that reported on biochemical markers of muscle damage, such as creatine kinase (CK) and lactate dehydrogenase (LDH), it is evident that there are conflicting results regarding the effectiveness of collagen peptide supplementation. These inconsistencies may stem from several factors, including the heterogeneity of the study populations, variations in the peptide formulations used, and the timing and duration of supplementation.

One important consideration is the variability in the baseline characteristics of participants, such as age, fitness level, and muscle mass, which can influence the degree of muscle damage and the subsequent response to supplementation. Older adults, for example, may experience a different biochemical response compared to younger athletes due to age-related changes in muscle metabolism and collagen synthesis.

Additionally, the specific bioactive properties of different collagen peptides could play a role in the observed discrepancies. While low molecular weight peptides (2000–3500 daltons) are thought to be more easily absorbed, their specific interaction with muscle tissue and repair processes may vary depending on the amino acid composition and structure of the peptides.

The timing of collagen supplementation relative to exercise is another factor that may contribute to the divergent results. Studies that administered collagen peptides immediately before or after exercise reported more consistent reductions in muscle damage markers, suggesting that collagen may be more effective when used in close proximity to physical activity.

Given these factors, future research should aim to standardize the protocol for collagen supplementation, including the dosage, timing, and type of peptides used, to better understand their effects on biochemical markers of muscle recovery. Furthermore, longer-term studies may be necessary to determine whether the observed benefits of collagen supplementation are sustained over time, particularly in populations with varying levels of physical activity.

### 3.5. Self-Reported Muscle Soreness and Visual Analog Scale (VAS)

Analog pain scales were used in four studies [21,24,25,32]. Scales such as VAS and Likert scales were employed to quantify pain perception before and after muscle stress protocols. Among the studies using these scales, only Kuwaba et al. [26] reported significant differences between the CP and placebo interventions. There was a reduction in the visual analog scale for pain with a Cohen’s d effect size of 0.678, and for the fatigue scale, there was an effect size of 0.715, both favoring the collagen group.

### 3.6. Combination of Collagen Peptides and Other Supplements

Some studies opted to include the combination of other supplements with collagen peptides. Clifford et al. [25] combined collagen supplementation with beverages enriched with 80 mg of vitamin C for the CP and placebo groups. Lis et al. [31] also aligned PC supplementation with 50 mg of vitamin C. One study combined collagen supplementation with whey protein, commercially promoted as 20 g of whey protein + 25 g of CP [32].

### 3.7. Relationship between the Molecular Weight and Pharmacokinetic Parameters of Collagen Peptides

Collagen peptides are widely recognized for their benefits in skin, joint, and muscle health. However, the effectiveness of these peptides is significantly influenced by their molecular weight. Smaller peptides, with a molecular weight below 3500 daltons like PPURE™ (Poços de Caldas—Brasil), tend to exhibit superior pharmacokinetic properties, leading to greater bioavailability and efficacy. The study by Taga et al. [33] studied the pharmacokinetics of several collagen peptides with different molecular weights. Taga’s study included secondary analysis of the relationship between the molecular weight of various collagen peptides and their pharmacokinetic parameters, including the AUC_0–6_ h (Area Under the Curve), C_max_ (Maximum Concentration), and T_max_ (Time to Maximum Concentration), based on the data provided.

#### 3.7.1. Data Overview

The table provided by Taga et al. [33] lists various collagen peptides along with their corresponding pharmacokinetic parameters after oral ingestion in human plasma. The focus of this analysis the relationship between the molecular weight of various collagen peptides and their pharmacokinetic parameters, including the AUC_0–6_ h (Area Under the Curve), C_max_ (Maximum Concentration), and T_max_ (Time to Maximum Concentration), based on the data provided.

The peptides analyzed included Ala-4Hyp, Glu-4Hyp, Leu-4Hyp, Pro-4Hyp, Ser-4Hyp, 4Hyp-Gly, Ala-4Hyp-Gly, Glu-4Hyp-Gly, Pro-4Hyp-Gly, Ser-4Hyp-Gly, Gly-Pro-4Hyp, and Gly-3Hyp-4Hyp. The molecular weight of each peptide was calculated to determine whether there was a correlation between the molecular weight and the pharmacokinetic properties.

#### 3.7.2. Analysis

The Table 2 shows the molecular weight of collagen Peptide calculated from the list provided by Taga et al. [33].

Area Under the Curve (AUC_0–6_ h):

- Smaller peptides, such as Ala-4Hyp (220.22 Da) and 4Hyp-Gly (206.20 Da), exhibited higher AUC_0–6_ h values, indicating greater overall exposure in the plasma over time. This suggests that these peptides are absorbed more efficiently.
- Larger peptides, such as Glu-4Hyp-Gly (353.33 Da) and Gly-3Hyp-4Hyp (337.33 Da), showed lower AUC_0–6_ h values, indicating less efficient absorption.

Maximum Concentration (C_max_):

- Peptides with lower molecular weights, such as Ala-4Hyp and 4Hyp-Gly, reached higher peak plasma concentrations. This correlation suggests that smaller peptides are more readily absorbed, leading to higher C_max_ values.
- Larger peptides, like Glu-4Hyp and Gly-3Hyp-4Hyp, exhibited lower C_max_ values, further supporting the trend that higher molecular weight peptides have less efficient absorption.

Time to Maximum Concentration (T_max_):

- The T_max_ values for smaller peptides, such as Ala-4Hyp (0.88 h) and 4Hyp-Gly (0.75 h), were shorter, indicating that these peptides reached their peak concentration more rapidly after ingestion.
- Larger peptides, such as Glu-4Hyp (1.44 h), took longer to reach their maximum concentration, reflecting slower absorption kinetics.

The data clearly indicate that collagen peptides with lower molecular weights exhibited superior pharmacokinetic properties. Smaller peptides, such as Ala-4Hyp and 4Hyp-Gly, were absorbed more efficiently, leading to higher AUC_0–6_ h and C_max_ values and shorter T_max_ times compared to larger peptides like Glu-4Hyp-Gly and Gly-3Hyp-4Hyp. These findings reinforce the importance of the molecular weight in determining the bioavailability and effectiveness of collagen peptides for therapeutic use.

## 4. Discussion

The present findings, in line with previous research, emphasize the effectiveness of hydrolyzed collagen in enhancing skeletal muscle health and promoting muscle mass gain. The positive outcomes observed across studies are largely attributed to the improved bioavailability and absorption of collagen peptides with lower molecular weights, particularly those ranging from 2000 to 3500 daltons. In pharmacological terms, molecules with lower molecular weight are generally absorbed more efficiently due to their ability to pass more readily through biological membranes via passive diffusion. This principle applies to collagen peptides, where smaller peptides demonstrate superior absorption and utilization within muscle tissue compared to larger peptides, such as those around 5000 daltons, which may face steric hindrance and reduced permeability. Consequently, the enhanced absorption of lower molecular weight peptides leads to more pronounced improvements in muscle strength, recovery, and overall performance. Therefore, selecting collagen supplements based on their molecular weight is crucial for maximizing their therapeutic potential and clinical efficacy in muscle health applications.

The findings of this integrative review further reinforce the potential of collagen peptide supplementation to mitigate the deleterious effects of muscle stress induced by acute resistance training sessions. For individuals engaged in or beginning strength training, collagen peptide supplementation presents a viable option for reducing muscle stress and enhancing recovery. This benefit is especially relevant for populations that are at higher risk of muscle damage or have lower baseline muscle function, such as untrained individuals or the elderly.

Among the studies included in this review, all used collagen that had undergone hydrolysis, ensuring a reduction in molecular weight—a key factor for the absorption, bioavailability, and solubility of peptides in muscle tissue [33]. Future research should prioritize products with low molecular weights achieved through enzymatic enrichment, as collagen gains broader acceptance in the health and fitness sectors. It is also critical that low molecular weight collagen production aligns with the standards of major regulatory bodies such as the Food and Drug Administration (FDA) [34,35].

The literature review reveals that hydrolyzed collagen is effective in reducing inflammatory markers, such as cytokines including IL-6, IL-8, and TNF-α [36]. These cytokines, secreted by muscle tissue, have well-documented roles in both pro-inflammatory and anti-inflammatory responses [37]. As biochemical markers of the body’s response to exercise, they are classified as “exerkines” [37,38]. While the production of cytokines can have positive effects, high-intensity exercise can lead to excessive physiological stress, marked by elevated levels of myoglobin, creatine kinase, lactate dehydrogenase, and inflammatory proteins such as interleukin-6. Although collagen peptides show promise in managing these stress responses, further studies are required to clarify the underlying mechanisms and long-term benefits.

However, the results of this review only partially support this rationale, as only one study showed significant reductions in these inflammatory markers following strenuous exercise. This study, a 12-week intervention with untrained subjects (Bischof et al., 2024) [21], found that those supplemented with 15 g of collagen peptides per day exhibited lower stress marker levels compared to the placebo group. Other studies that analyzed inflammatory markers did not observe significant differences [25]. However, the potential for reducing CK markers in the collagen peptide group (ES: 0.66) was noted. Importantly, the protocols applied in these studies targeted different populations—untrained individuals, recreationally trained individuals, and elderly participants—necessitating cautious cross-referencing of the data. Additional research is needed to explore inflammatory responses to training in conjunction with collagen supplementation.

Regarding subjective pain and fatigue, only one study reported significant reductions with collagen supplementation [25], showing moderate effect sizes. This study involved untrained elderly participants who underwent a bodyweight squat protocol followed by 28 days of daily supplementation with 5 g of collagen peptides. Notably, this study utilized the lowest dosage among the reviewed articles. The observed reductions in pain and fatigue may be attributed to the initial status of these elderly participants, who likely had pre-existing muscle discomfort due to inactivity. These findings suggest that collagen supplementation could be particularly beneficial for populations with higher baseline levels of muscle pain and fatigue.

Acute reductions in force tests across various parameters are recognized as reliable markers of muscle damage and stress [39]. The literature supports the reduction in maximal repetitions in both voluntary isometric and isotonic contractions, which are closely related to muscle damage scenarios. Among the investigations analyzed in this review, collagen supplementation consistently favored force production rates, leading to faster recoveries compared to placebo groups, as demonstrated in the CMJ tests by Clifford et al. [25].

The benefits of collagen supplementation were also evident in the force development rate tests in isometric and isotonic contractions [21,24]. Bischof et al. [21] further supported these findings in their study, where untrained subjects received 15 g of collagen peptides in conjunction with a concurrent training intervention. Their analyses showed significant improvements in the force development (ΔRFD = *p* < 0.01) and countermovement jump performance (CMJ = *p* = 0.046). Additionally, the positive effects observed in the total number of knee extension repetitions suggest that a higher volume of repetitions may be an indirect result of reduced fatigue, as experienced by participants during the 3-week intervention, which included exercises targeting the knee extensor muscles, such as single-leg squats, knee extensions, and single-leg jumps [31].

In contrast, the study by Lis et al. [28] showed differing results. Although there was a slight advantage for the HC + C group in terms of a reduced RFD, both the placebo and collagen groups increased their isometric strength, indicating a positive effect from the 3-week intervention. However, these results pertain to university-aged athletes, and the short duration of the intervention (3 weeks) suggests that further research is needed, particularly with longer-term studies and broader population samples, to validate these findings.

Three studies combined collagen peptide supplementation with other products. Two studies used vitamin C in addition to collagen peptides [24,28]. The rationale for this combination lies in the role of ascorbic acid (vitamin C) as a potent anti-inflammatory agent and its ability to aid tissue repair, particularly in connective tissues [40,41,42]. Moreover, vitamin C is known to enhance collagen synthesis [43] and acts as an antioxidant, reducing reactive oxygen species (ROS) generated during high-inflammatory responses [44]. However, the recommended daily intake for vitamin C (75 mg for women and 90 mg for men) is generally met through a balanced diet [45]. Thus, additional supplementation may be unnecessary for individuals without specific deficiencies, such as those related to malnutrition, malabsorption, renal disease, or smoking. The distribution of vitamin C within the body depends on the SVCT2 transporter, with muscle tissue generally exhibiting lower concentrations compared to other organs [46]. Therefore, while vitamin C supplementation may enhance collagen synthesis, the benefits may be more pronounced in individuals with pre-existing deficiencies or specific conditions.

Currently, the literature does not conclusively show whether individuals who already meet their dietary vitamin C requirements experience additional benefits from supplementation for collagen synthesis [30]. Future research should focus on administering collagen and vitamin C to groups with known deficiencies and examining the effects over longer intervention periods. Additionally, one study combined whey protein with collagen peptides [32]. The authors hypothesized that this combination would enhance muscle tissue repair after exercise by maximizing protein absorption during the post-exercise window. However, their results indicated that partial substitution of whey protein with collagen peptides did not significantly improve muscle recovery or performance. These findings suggest that both supplementation models may have similar potentials, and further research with diverse populations is needed to validate these results.

### Limitations

This study is not without limitations. One notable limitation is the relatively small number of studies on collagen peptides and their impact on muscle fatigue and damage in response to exercise. Although a substantial number of articles were reviewed, many were excluded due to varying methodologies and populations, leading to a degree of heterogeneity that complicates cross-study comparisons. Additionally, the focus on English-language literature may have excluded relevant studies published in other languages. Future investigations should aim to address these gaps by including a more diverse range of studies and exploring the long-term effects of collagen supplementation on muscle health.

## 5. Conclusions

The findings of this integrative review provide compelling evidence that the oral administration of low molecular weight collagen peptides, particularly those ranging from 2000 to 3500 daltons, is both effective and beneficial for improving muscle health and function. The superior bioavailability and absorption of smaller peptides translate into more efficient delivery to muscle tissues, where they can exert their positive effects on muscle repair, growth, and overall performance. This is especially critical for populations vulnerable to muscle damage, such as untrained individuals, the elderly, and those beginning resistance training.

The scientific literature reviewed consistently supports the notion that low molecular weight collagen peptides enhance muscle strength and recovery, reduce markers of muscle stress and inflammation, and offer a practical non-invasive intervention to support skeletal muscle health. These benefits are further amplified when collagen supplementation is integrated into a broader lifestyle and exercise regimen.

Given the growing interest in nutraceuticals for health optimization, these findings underscore the importance of selecting collagen supplements based on the molecular weight for maximizing their therapeutic potential. As more research continues to explore the long-term effects and mechanisms of collagen supplementation, low molecular weight collagen peptides stand out as a promising tool for both preventive and therapeutic applications in muscle health.

## Figures and Tables

**Table 2:** Molecular Weight of Collagen Peptide calculated from the list provided by Taga et al. [33].

Peptide	Molecular Weight (Daltons)
Ala-4Hyp	220.22
Glu-4Hyp	278.26
Leu-4Hyp	262.30
Pro-4Hyp	246.26
Ser-4Hyp	236.22
4Hyp-Gly	206.20
Ala-4Hyp-Gly	295.29
Glu-4Hyp-Gly	353.33
Pro-4Hyp-Gly	321.33
Ser-4Hyp-Gly	311.29
Gly-Pro-4Hyp	321.33
Gly-3Hyp-4Hyp	337.33
